# Corticosteroid resistance in sepsis is influenced by microRNA-124-induced downregulation of glucocorticoid receptor-α

**DOI:** 10.1186/cc11786

**Published:** 2012-11-14

**Authors:** P Möhnle, C Ledderose, J Briegel, S Kreth

**Affiliations:** 1 Ludwig-Maximilians-Universität München, Klinik für Anaesthesiologie, München, Germany; 2 Universitätsklinikum Mannheim, Klinik für Anästhesiologie und Intensivmedizin, Mannheim, Germany

## Background

Acquired glucocorticoid resistance frequently complicates the therapy of sepsis. It leads to an exaggerated proinflammatory response and has been related to altered expression profiles of glucocorticoid receptor isoforms glucocorticoid receptor-α (mediating anti-inflammatory effects) and glucocorticoid receptor-β (acting as a dominant negative inhibitor). We investigated the impact of glucocorticoid receptor isoforms on glucocorticoid effects in human T cells. We hypothesized that changes of the ratio of glucocorticoid receptor isoforms impact glucocorticoid resistance and that glucocorticoid receptor-α expression is controlled by microRNA-mediated gene silencing.

## Methods

First, T cells from healthy volunteers (native and CD3/CD28-stimulated cells with or without addition of hydrocortisone) were analyzed for the expression of glucocorticoid receptor isoforms by quantitative PCR. Additionally, effects of gene silencing of glucocorticoid receptor-β by siRNA transfection were determined. Secondly, microRNA-mediated silencing was evaluated by cloning of a glucocorticoid receptor-α-specific 3'-untranslated-region reporter construct and subsequent transfection experiments in cell cultures. Effects of miRNA transfection on glucocorticoid receptor-α expression were analyzed in Jurkat T cells and in T cells from healthy volunteers (quantitative PCR and western blotting). Finally, expression of glucocorticoid receptor-α, glucocorticoid receptor-β, and miR-124 was tested in T cells of sepsis patients (*n *= 24).

## Results

Stimulation of T cells induced a significant upregulation of glucocorticoid receptor-α (not glucocorticoid receptor-β), thereby possibly rendering T cells more sensitive to glucocorticoids; this T-cell response was hindered by hydrocortisone. Silencing of glucocorticoid receptor-β doubled the inhibitory effects of glucocorticoids on IL-2 production. MicroRNA-124 was proved to specifically downregulate glucocorticoid receptor-α. Furthermore, a glucocorticoid-induced threefold upregulation of microRNA-124 was found. T cells of sepsis patients exhibited slightly decreased glucocorticoid receptor-α and slightly increased miR-124 expression levels, whereas glucocorticoid receptor-β expression was twofold upregulated (*P *< 0.01) and exhibited a remarkable interindividual variability.

## Conclusion

Glucocorticoid treatment induces expression of miR-124, which downregulates glucocorticoid receptor-α thereby limiting anti-inflammatory effects of glucocorticoids. Steroid treatment might aggravate glucocorticoid resistance in patients with high glucocorticoid receptor-β levels.

